# Attribution of *Salmonella enterica* to Food Sources by Using Whole-Genome Sequencing Data

**DOI:** 10.3201/eid3104.241172

**Published:** 2025-04

**Authors:** Erica Billig Rose, Molly K. Steele, Beth Tolar, James Pettengill, Michael Batz, Michael Bazaco, Berhanu Tameru, Zhaohui Cui, Rebecca L. Lindsey, Mustafa Simmons, Jess Chen, Drew Posny, Heather Carleton, Beau B. Bruce

**Affiliations:** Centers for Disease Control and Prevention Atlanta, Georgia, USA (E.B. Rose, M.K. Steele, B. Tolar, Z. Cui, R.L. Lindsey, J. Chen, H. Carleton, B.B. Bruce); US Food and Drug Administration, College Park, Maryland, USA (J. Pettengill, M. Batz, M. Bazaco); US Department of Agriculture, Washington, DC, USA (B. Tameru, M. Simmons, D. Posny)

**Keywords:** Salmonella enterica, bacteria, source attribution, foodborne illness, food safety, random forest model, whole-genome sequence data

## Abstract

*Salmonella enterica* bacteria are a leading cause of foodborne illness in the United States; however, most *Salmonella* illnesses are not associated with known outbreaks, and predicting the source of sporadic illnesses remains a challenge. We used a supervised random forest model to determine the most likely sources responsible for human salmonellosis cases in the United States. We trained the model by using whole-genome multilocus sequence typing data from 18,661 *Salmonella* isolates from collected single food sources and used feature selection to determine the subset of loci most influential for prediction. The overall out-of-bag accuracy of the trained model was 91%; the highest prediction accuracy was for chicken (97%). We applied the trained model to 6,470 isolates from humans with unknown exposure to predict the source of infection. Our model predicted that >33% of the human-derived *Salmonella* isolates originated from chicken and 27% were from vegetables.

*Salmonella enterica* bacteria are a leading cause of foodborne illness and hospitalization in the United States (*1*). Improving our understanding of the sources and transmission vehicles of salmonellosis can guide prevention strategies and policy measures to reduce the prevalence of disease attributable to this pathogen. Exposure to *Salmonella* bacteria can occur through food, drinking water, animal contact, environmental sources such as soil and water, and infected persons (*2*). Some *Salmonella* serotypes are highly host-specific. For example, *S. enterica* serovar Dublin is primarily associated with infections in cattle and sheep, whereas serovar Gallinarum is almost exclusively associated with infections in poultry. In contrast, other serotypes, such as serovars Enteritidis and Typhimurium, are associated with infection of a wider range of human and animal hosts (*3*–*5*). Most *Salmonella* infections in the United States are most likely transmitted through food, especially the commonly observed serotypes, including *Salmonella* Enteritidis, 4,[5],12:i:-, Newport, Javiana, and Typhimurium (*1*,*2*).

Source attribution is the process used to ascribe human infections to specific sources. Attribution estimates can be made for different points in the farm-to-fork continuum, depending on the data and methods used, which can affect interpretation of results. Outbreak-based attribution estimates reflect foods as consumed and might not reflect risks at other points in the farm-to-fork continuum. Data from foodborne outbreaks often are used to estimate the relative contribution of food sources to an attribution estimate for all human infection (both sporadic infections and infections linked to outbreaks) (*6*). However, only 5% of *Salmonella* illnesses can be linked to known outbreaks, so these analyses might not be representative of the risks associated with sporadic (e.g., nonoutbreak) illnesses (*7*).

Other methods can attribute illnesses at points in the continuum before consumption, such as using microbial subtyping to link clinical isolates from sporadic human cases to isolates from food, animals, and other potential sources (*8*,*9*). Until recently, most of these analyses were based on traditional laboratory typing methods. With the advent of whole-genome sequencing (WGS) as a subtyping method, developing source prediction models that are based on a more discriminatory subtyping method with publicly available surveillance data has the potential to clarify the source of *Salmonella* infections and improve attribution estimates.

Machine learning algorithms that use WGS have been shown to successfully attribute human infections to sources (*10*–*14*); however, those studies were based on a small number of isolates and serotypes. For our study, we applied a random forest machine learning classifier algorithm to determine the most likely sources responsible for human salmonellosis cases in the United States overall and for serotypes commonly associated with illness. Random forest algorithms are a supervised machine learning classification method that use highly dimensional data (i.e., many predictor variables) to predict membership to a predetermined set of categories. We developed a random forest model by using WGS data of *Salmonella* isolates with known food sources to estimate the distribution of sources of human *Salmonella* infections.

## Methods

### WGS Data from Known Food Sources for Model Training

We compiled all available *Salmonella* isolates collected from food or cecal samples collected from food animals at slaughter in the National Center for Biotechnology Information (NCBI) database (accessed in 2018) and publicly available metadata, plus additional metadata available from the US Department of Agriculture’s Food Safety and Inspection Service (USDA-FSIS), Food and Drug Administration (FDA), and Centers for Disease Control and Prevention (CDC). We then manually identified isolates that could be definitively categorized into 1 of 15 analytical categories in the Interagency Food Safety Analytics Collaboration (IFSAC) scheme (https://www.cdc.gov/ifsac/php/projects/food-categorization-scheme.html) (*15*). Those categories included beef, chicken, dairy, eggs, pork, nuts/seeds, fruit, shellfish, fish, turkey, vegetables, grains/beans, game, other poultry, and other meat. Isolates from dishes or foods with ingredients in multiple categories were excluded (e.g., lasagna). Because most *Salmonella* isolates available in NCBI were collected from chicken, we randomly selected a subset of 50% of those chicken isolates to mitigate class imbalance. To further mitigate the effects of class imbalance, we used inverse class weighting to build the model. To ensure our dataset did not contain duplicated isolates, we only included isolates whose sequence read archive identification number was found in NCBI’s Pathogen Browser. In addition, if multiple isolates were associated with the same strain (i.e., specimen) identifier, we removed those isolates from the dataset. This process resulted in a total of 18,661 isolates (Table 1).

**Table 1:** Number of *Salmonella* isolates used to train a random forest model by food commodity, location of collection (domestic vs. international sampling) and, if domestic, entity that collected the isolate, 2003–2018*†

Category	Domestic sampling‡					International§	Missing location	Total
	FSIS	NARMS	FDA/CFSAN	Other	Total			
Chicken	1,477	1,010	3,075	31	5,593	168	72	5,833 (31)
Vegetables	NA	NA	1,284	42	1,326	1,055	9	2,390 (13)
Turkey	121	486	1,630	33	2,270	6	12	2,288 (12)
Pork	362	1,226	238	67	1,893	121	12	2,026 (11)
Beef	344	597	628	34	1,603	77	3	1,683 (9)
Fish	13	NA	47	1	61	984	2	1,047 (6)
Nuts/seeds	NA	NA	415	4	419	516	12	947 (5)
Fruit	NA	NA	151	9	160	532	6	698 (4)
Shellfish	NA	NA	20	NA	20	527	10	557 (3)
Egg	266	NA	60	15	341	32	11	384 (2)
Other poultry	52	NA	43	13	108	94	3	205 (1)
Other meat	NA	NA	80	2	82	112	2	196 (1)
Grains/beans	NA	NA	124	NA	124	40	6	170 (1)
Dairy	NA	NA	74	7	81	71	7	159 (1)
Game	NA	NA	63	NA	63	15	NA	78 (<1)
Total	2,635 (14)	3,319 (18)	7,932 (43)	258 (1)	14,144	4,350 (23)	167 (<1)	18,661

*Values are no. or no. (%). CDC, Centers for Disease Control and Prevention; CFSAN, Center for Food Safety and Applied Nutrition; FDA, Food and Drug Administration; FSIS, Food Safety Inspection Service; HACCP, Hazard Analysis and Critical Control Point; NA, not available; NARMS, National Antimicrobial Resistance Monitoring System; USDA–ARS, US Department of Agriculture’s Agricultural Research Service. †Includes 603 isolates collected prior to 2003. ‡Domestic sampling by various agencies and programs: FSIS (HACCP, baselines, exploratory), NARMS (FSIS and FDA), FDA CFSAN (including GenomeTrakr Network), and other (CDC PulseNet, USDA–ARS, university laboratories, and state laboratories). §International includes isolates from nondomestic GenomeTrakr laboratories.

### WGS Data from Human Infections with Unknown Source

We collected *Salmonella* isolates submitted to the Foodborne Diseases Active Surveillance Network (FoodNet) during 2014–2017 with unknown source of illness and no history of international travel for the affected patient. FoodNet conducts population-based active surveillance for laboratory-confirmed enteric infections, including those caused by *Salmonella*, and has a catchment covering ≈15% of the US population (*16*). During 2014–2017, data were collected at 10 sites: Connecticut, Georgia, Maryland, Minnesota, New Mexico, Oregon, Tennessee, and select counties in California, Colorado, and New York. FoodNet collects data on patient demographics, clinical information, outbreak association, and international travel history in the 7 days before illness onset. Since 2014, FoodNet also has collected data on various exposures, including consuming meats, fruits, and vegetables, during the 7 days before illness onset.

### Whole-Genome Multilocus Sequence Typing Data

We assembled food isolate sequences in SPAdes, then analyzed them in BioNumerics version 7.6 (bioMérieux, https://www.biomerieux.com) to assign assembly based allele calls in the *Salmonella* whole-genome multilocus sequence typing (wgMLST) scheme (*17*). We assembled human isolate sequences by using SPAdes (https://github.com/ablab/spades), and we generated allele calls from the PulseNet *Salmonella* wgMLST allele database. Given the time over which the sequence data were generated, we used multiple versions of SPAdes to account for upgrades in the software, the oldest version being SPAdes version 3.7.1. We analyzed the annotated data by using BioNumerics version 7.6. We generated serotype data for each sequence by using SeqSero2 (https://github.com/denglab/SeqSero2). We described results for *Salmonella* overall and by common serotypes, including *Salmonella* Enteritidis, Typhimurium, Javiana, Newport, Infantis, Heidelberg, and 4,5,[12]:i:-.

### Random Forest Algorithm

We trained a random forest model by using wgMLST data on isolates from 15 known single food categories (*18*). We excluded loci that were missing allele call information in >99% of isolates. Otherwise, we treated a missing allele as a unique nominal value. We defined tree splits once by using the first principal component of the weighted covariance matrix, which is computationally efficient for nominal features with a large number of levels (*19*). We evaluated feature importance (i.e., how informative each locus was for accurately predicting food source) by using permutation importance on 2-fold cross-validation (*20*). We calculated the relative importance of each feature and then ran the random forest with inverse class weighting and 1,000 trees 20 times on a stratified random 75–25 train-test split of the data by using the top-10 loci. We calculated the mean accuracy and κ of the test data by using this top set of features. We repeated this approach, increasing the top set of loci by 50 each time, and finally with all loci present in >1% of isolates. We then determined an optimal model as the one with maximum median accuracy and κ. We assessed the confusion matrix and accuracy for specific common serotypes of interest on the optimal model. We then assessed out-of-bag accuracy overall and by food category.

We applied the trained model to human isolates of *Salmonella* with an unknown source. For each isolate, if the maximum predicted probability for a food category was >0.50, we assigned the isolate that category as the likely source. If the largest predicted probability was <0.50, we assigned the isolate a class of unknown or nonfood source. We then renormalized the distribution of predictions among human isolates assigned to a category, so the predicted percentages of isolates from each of the 15 categories totaled 100%. We estimated the distribution of sources of isolates from human illnesses overall and by the common serotypes, including *Salmonella* Enteritidis, Typhimurium, Javiana, Newport, Infantis, Heidelberg, and 4,5,[12]:i:-.

We performed all analyses in R version 4.1.1 (The R Project for Statistical Computing, https://www.r-project.org). We developed the random forest model by using the ranger package (*21*).

## Results

### WGS Data from Known Food Sources for Model Training

We trained the random forest model on 18,661 isolates, of which 16,756 (89.7%) were single food sources and 1,905 (10.2%) were cecal samples from food animals (Appendix 1 Table 1). In this analysis, we assumed the cecal isolates were representative of a food source. We included isolates from all over the world; however, most isolates (76%) were collected in the United States. Among isolates with a known year of collection, 603 isolates (3.2%) were collected before 2003, 8,409 (45.3%) were collected during 2003–2013, 9,038 (48.7%) were collected during 2014–2017, and 505 (2.7%) were collected after 2017. Sample collection year was not available for 106 isolates (0.6%). 

**Figure 1:**
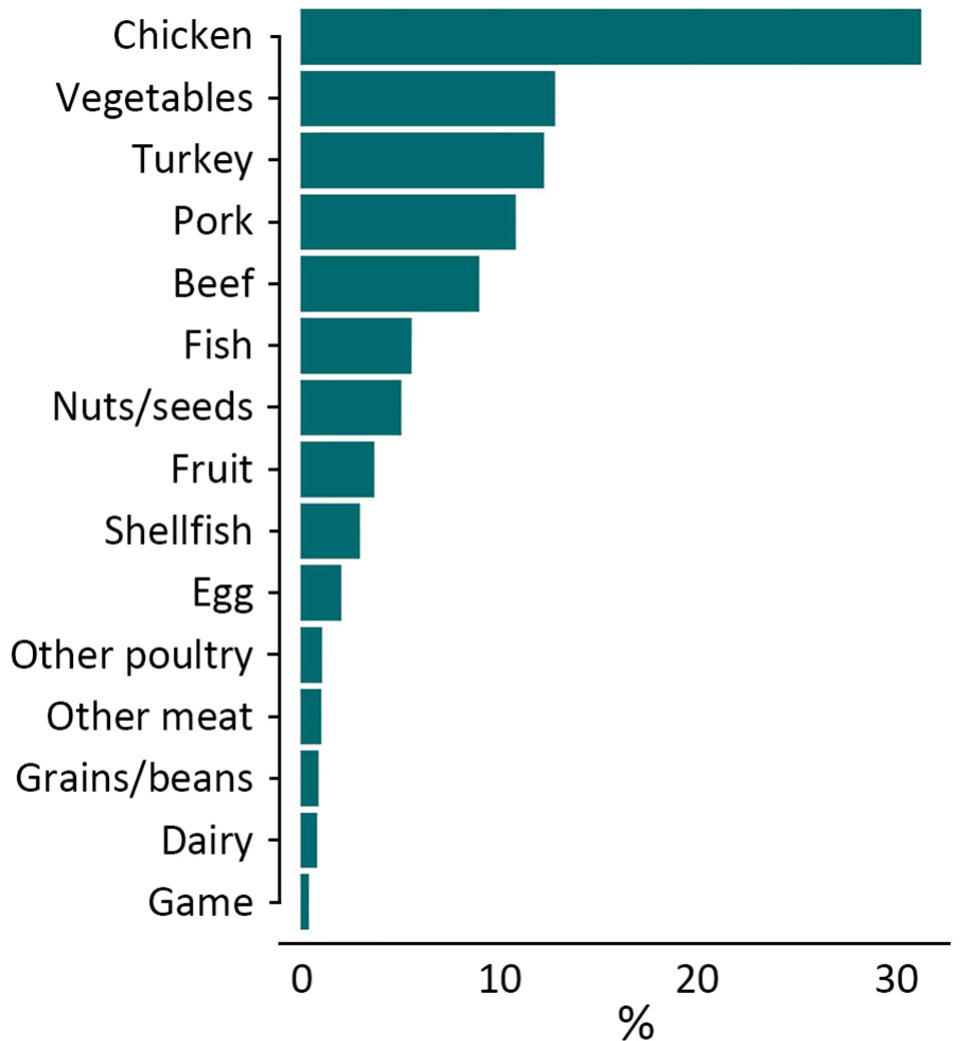
Percentage of *Salmonella* isolates collected from known single source foods in the United States and other countries from 2003–2018 (used as training data in random forest model), by food category (N = 18,661, including 613 isolates collected before 2003).

**Figure 2:**
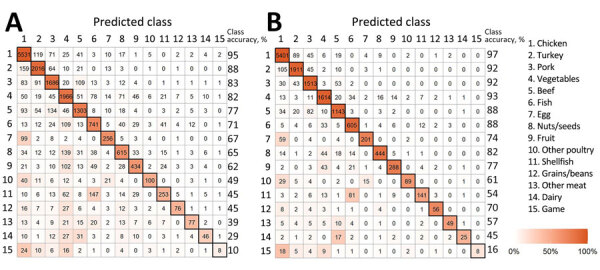
Confusion matrix from the random forest model trained on *Salmonella* isolates collected from single food categories in the United States and other countries during 2003–2018 and 613 isolates collected before 2003. A) Confusion matrix for all *Salmonella* isolates from single food categories (N = 18,661). B) Confusion matrix from the random forest model for *Salmonella* isolates from single food categories with a maximum predictive probability of >0.50 (n = 14,888).

Chicken was the most common food source (n = 5,833 [31.3%]), followed by vegetables (n = 2,390 [12.8%]), turkey (n = 2,288 [12.3%]), and pork (n = 2,026 [10.9%]) (Figure 1). We accounted for the skewed distribution observed in the training data across 15 categories during the random forest model development by using inverse class weighting, a cost-sensitive approach to classification on imbalanced data. The most common serotypes were *Salmonella* Kentucky (n = 1,604 [8.6%]), Typhimurium (n = 1,539 [8.2%]), Enteritidis (n = 1,311 [7.0%]), and Heidelberg (n = 1,280 [6.9%]) (Appendix 2 Figure 1). A total of 22,457 chromosomal loci were present, including 3,002 core loci and 8,143 loci that were present in >1% of isolates (Appendix 2 Figure 2). Among those loci, the number of unique alleles within a locus (that also were missing as a unique value) ranged from 2 to 5,509. The median number of unique alleles was 134 (interquartile range 28–465).

### WGS Data from Human Infections with Unknown Source

For food source prediction of human isolates, we used 6,470 isolates collected from humans in cases where the source of illness was unknown and no history of international travel was reported. Isolates were collected during 2014–2017; a total of 280 isolates (4.3%) were collected in 2014, 833 (12.9%) were collected in 2015, 2,429 (37.5%) were collected in 2016, and 2,928 (45.2%) were collected in 2017. Most isolates were not associated with an outbreak (88%). The most common serotypes among the human isolates were *Salmonella* Enteritidis (n = 1,446 [22.3%]), Typhimurium (n = 716 [11.1%]), Newport (n = 706 [10.9%]), Javiana (n = 553 [8.5%]), and 4,[5],12:i:- (n = 416 [6.4%]) (Appendix 2 Figure 1).

### Permutation Importance and Model Performance

**Figure 3:**
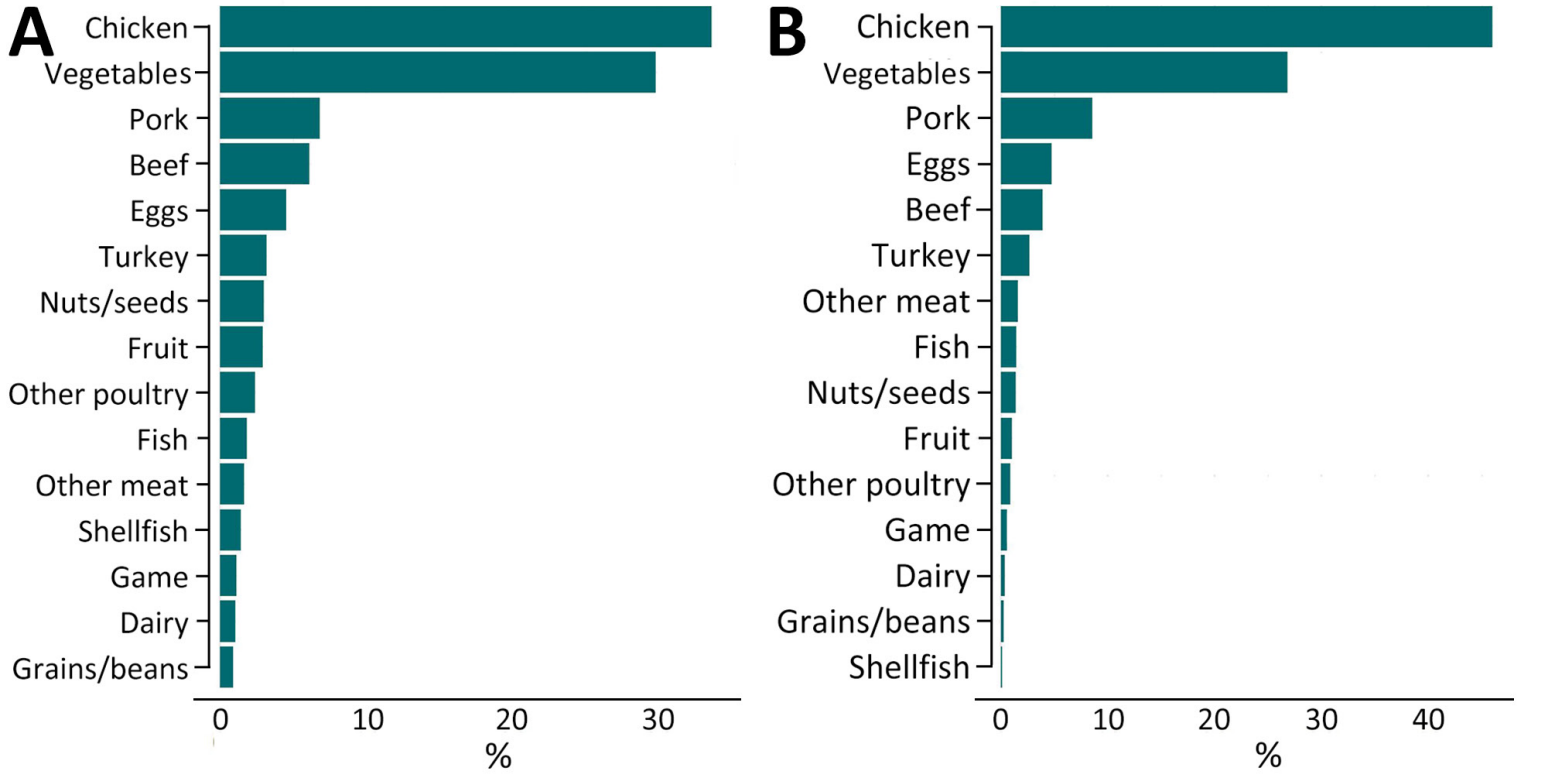
Predictions from a random forest model of sources of US human illnesses among patients without reported history of international travel who had whole-genome sequenced *Salmonella* isolates reported to the Foodborne Diseases Active Surveillance Network, 2014–2017. A) Predictions of sources of human illnesses among all patients (N = 6,470). B) Predictions of food category sources of human illnesses among patients renormalized among isolates with a >0.50 probability of attribution to a single food category (n = 3,686).

The accuracy of the median test data model, when applied to a stratified 75–25 split of the food data with permutation importance and inverse class weighting, ranged from 0.51 using the top-10 loci to 0.74 using the top-7,360 loci (Appendix 2 Figure 3). Of these 7,360 loci, 2,987 were core (Appendix 1 Table 2). The median κ was maximized at 0.70 using the top 7,360 loci; we selected that model as the optimal model.

The confusion matrix displays the accuracy among each class and patterns of misclassification among training isolates using out-of-bag estimates (Figure 2, panel A; Appendix 2 Figure 4, panel A). The overall accuracy of the model using all isolate predictions was 0.81 (Table 2). The model performed best for chicken (0.95 out-of-bag accuracy) and also performed well for other common sources of *Salmonella*, such as turkey (0.88), pork (0.83), vegetables (0.82), and beef (0.77). The model had lowest accuracy for less common sources; those with <0.40 accuracy included game (0.10), dairy (0.29), and other meat (0.39).

**Table 2:** Out-of-bag and train-test performance statistics for a random forest model trained on *Salmonella* isolates collected from single food sources and using the top 7,360 loci determined from feature selection*

Characteristic	Out-of-bag	Train-test, 75–25†
Accuracy	0.81	0.74
κ	0.77	0.68
Balanced accuracy	0.61	0.52
AUC-ROC, uniform distribution‡	0.93	0.92
AUC-ROC, a priori distribution‡	0.97	0.95

*AUC-ROC, area under the receiver operating characteristic curve. †Indicates 75% of the data was used to train the model, whereas 25% was used to test the model. ‡Calculation described at https://www.math.ucdavis.edu/~saito/data/roc/ferri-class-perf-metrics.pdf.

When we assessed out-of-bag estimates while limiting analysis to the 14,888 isolates with a maximum predicted probability from a single class of >0.50, the overall accuracy increased to 0.91; we observed the highest accuracy (0.97) for chicken (Figure 2, panel B; Appendix 2 Figure 4, panel B). In addition, the accuracy of each class increased, and all class accuracies were >0.50 except for game (0.16) and dairy (0.45).

### Model Prediction of Human Data

Among all patients without a reported history of international travel and with unknown source of infection, the most common predicted sources of illness were chicken (n = 2,170 [34%]) and vegetables (n = 1,924 [30%]). All other sources accounted for <10% of illnesses (Figure 3, panel A). When we assigned human isolates with a maximum predicted probability of <0.50 as from an unknown source, 44% of isolates (n = 2,859) were labeled unknown. There is more confidence in the predictions with prediction probabilities >0.50 that are retained (i.e., not reclassified as unknown) and are likely to arise from majority classes (such as chicken or vegetables) as opposed to minority classes as observed in the training data (Appendix 2 Figure 5). Among isolates assigned to a known source, 46% (n = 1,694) were predicted to be from chicken, 27% (n = 987) were predicted to be from vegetables, and all other sources of illness accounted for <10% of illnesses (Figure 3, panel B). In addition, the most common predicted source of serotypes *Salmonella* Enteritidis, Typhimurium, Heidelberg, and Infantis was from chicken. The most common predicted source of *Salmonella* Javiana and Newport was vegetables, and the most common source of *Salmonella* 4,[5],12:i:- was pork (Appendix 1 Table 3).

## Discussion

Our study developed a random forest model by using *Salmonella* wgMLST data from food sources to estimate the likely source of sporadic human illnesses. When using all isolate predictions, the model had >74% accuracy when both out-of-bag and train-test validation methods were used. When we restricted analysis to isolates with prediction probabilities >0.50, the out-of-bag accuracy increased to 91%. Chicken and vegetables were the most common predicted sources of salmonellosis in the United States. This result is consistent with previous analyses; however, our analysis found chicken to be linked to a substantially higher percentage of illnesses than in recent attribution estimates for poultry products on the basis of outbreak data (46% vs. 17%) (*6*). There are numerous possible reasons for this difference. One possible explanation might be that outbreak data reflect foods as consumed (which can become contaminated from another food during preparation), whereas the data in this study are from earlier points in the farm-to-fork continuum. Another explanation might be that risks associated with outbreaks differ from those associated with sporadic infections. By using our single model, we were able to estimate the contributions of sources to human salmonellosis overall and for serotypes that commonly cause human illness. We found notable differences in attribution across serotypes; we found that chicken was the most common estimated source of *Salmonella* Enteritidis, Typhimurium, Heidelberg, and Infantis, pork was the most common source of *Salmonella* 4,[5],12:i:-, and vegetables were the most common source of *Salmonella* Javiana and Newport.

In our approach, we assigned human isolates from a single source that were attributed to a category with >0.50 probability to that food category. We classified human isolates with <0.50 probability of attribution to any single source as either attributed to an unknown food or a nonfood source. We chose a cutoff of 0.50 by examining the accuracy of predictions by probability, considering that not all illnesses arise through foodborne transmission, and for ease of explanation (i.e., those with >0.50 probability were more likely than not to have come from that source). This approach could be tailored to adjust the sensitivity and specificity of the model for different purposes.

Without this prediction threshold, we estimate that about two thirds of human salmonellosis are attributable to chicken (34%) or vegetables (30%). With the prediction threshold, we retain only confident model predictions and estimate chicken and vegetables as the source of roughly 73% of salmonellosis cases. Moreover, cases were attributed to all 15 modeled categories, indicating that illnesses likely arise from various sources, which is consistent with a recent report of foodborne illness attribution from the Interagency Food Safety Analytics Collaboration (*6*).

Since routine WGS on *Salmonella* isolates began in 2019, we are better able to develop and explore new approaches for source attribution estimation. Similar studies in Europe and Australia have used core-genome multilocus sequence typing (cgMLST) and single-nucleotide polymorphism data to attribute certain *Salmonella* serotypes to animal reservoirs. Results from a logit boost algorithm on cgMLST data from animals estimated 53% of *Salmonella* Typhimurium’s sources in Denmark with 93% accuracy (*13*). Results from a Bayesian source attribution model using *Salmonella* Typhimurium cgMLST data in England and Wales attributed 60% of human cases to pigs (*22*).

One limitation of our model is that the training data were imbalanced; chicken represented the clear majority class. Subsequently, we then randomly selected a 50% subset to reduce the dataset, and all minority class isolates (i.e., nonchicken sources), which had been filtered out, were reincorporated. As a result, the input data was less imbalanced because the majority chicken class was reduced to only 31%. We also used a cost-sensitive learning approach through inverse class weighting scheme to handle the remaining class imbalance, which applies a heavier penalty on misclassifying the minority classes during model development. Given those steps, our model still predicts chickens as the dominant source in the test dataset of human isolates (>31%). Access to and incorporation of more isolates from nonchicken food categories would likely strengthen the model’s accuracy and precision. In addition, healthcare-seeking behaviors, access to health services, and other characteristics of the FoodNet surveillance area population might not reflect those of the entire United States and might limit the generalizability of our findings. The human isolates included in this analysis were a subset of FoodNet isolates that had wgMLST analysis performed and might not have been equally distributed across the FoodNet sites, which might also limit generalizability. Not all salmonellosis is foodborne; recent estimates indicate that 66% of all salmonellosis cases are from direct consumption of a contaminated food item (foodborne), and we only included food isolates in our training data (*23*). Including additional training data from nonfood sources, such as water, wild and domesticated animals, and the environment, could expand our estimation to also predict nonfood sources of infection as well. Food animals can contaminate the environment and indirectly contaminate vegetables and fruits and potentially make humans sick. Further, our approach does not distinguish where in the farm-to-fork continuum exposure occurred, and those food sources are not necessarily tied to consumption. Consequently, our model ascribes isolates to sources that might not actually be the source of a foodborne exposure.

Overall, our model predicted chicken and vegetables are top sources of salmonellosis cases in the United States. This analysis highlights the utility of applying classification algorithms, such as random forests, to analyze genomic data for foodborne illness source attribution. With further research, models like ours could be leveraged with existing genomic surveillance systems to support source identification in outbreak investigations and to help inform regulatory priority setting processes.

Appendix 1 Additional data for attribution of *Salmonella enterica* to food sources by using whole-genome sequencing data.

Appendix 2 Additional information for attribution of *Salmonella enterica* to food sources by using whole-genome sequencing data.

## References

[1] Scallan E, Hoekstra RM, Angulo FJ, Tauxe RV, Widdowson MA, Roy SL, et al. Foodborne illness acquired in the United States—major pathogens. Emerg Infect Dis. 2011;17:7–15. 10.3201/eid1701.P11101 21192848 PMC3375761

[2] Beshearse E, Bruce BB, Nane GF, Cooke RM, Aspinall W, Hald T, et al. Attribution of illnesses transmitted by food and water to comprehensive transmission pathways using structured expert judgment, United States. Emerg Infect Dis. 2021;27:182–95. 33350907 10.3201/eid2701.200316 PMC7774530

[3] Kingsley RA, Bäumler AJ. Host adaptation and the emergence of infectious disease: the *Salmonella* paradigm. Mol Microbiol. 2000;36:1006–14. 10844686 10.1046/j.1365-2958.2000.01907.x

[4] Uzzau S, Brown DJ, Wallis T, Rubino S, Leori G, Bernard S, et al. Host adapted serotypes of *Salmonella enterica.* Epidemiol Infect. 2000;125:229–55. 11117946 10.1017/s0950268899004379 PMC2869595

[5] Rabsch W, Andrews HL, Kingsley RA, Prager R, Tschäpe H, Adams LG, et al. *Salmonella enterica* serotype Typhimurium and its host-adapted variants. Infect Immun. 2002;70:2249–55. 10.1128/IAI.70.5.2249-2255.2002 11953356 PMC127920

[6] Interagency Food Safety Analytics Collaboration. Foodborne illness source attribution estimates for 2020 for *Salmonella, Escherichia coli* O157, *Listeria monocytogenes*, and *Campylobacter* using multi-year outbreak surveillance data, United States. 2022 [cited 2023 Oct 19]. https://www.cdc.gov/ifsac/media/pdfs/P19-2020-report-TriAgency-508.pdf

[7] Ebel ED, Williams MS, Cole D, Travis CC, Klontz KC, Golden NJ, et al. Comparing characteristics of sporadic and outbreak-associated foodborne illnesses, United States, 2004–2011. Emerg Infect Dis. 2016;22:1193–200. 10.3201/eid2207.150833 27314510 PMC4918141

[8] Pires SM, Vieira AR, Hald T, Cole D. Source attribution of human salmonellosis: an overview of methods and estimates. Foodborne Pathog Dis. 2014;11:667–76. 10.1089/fpd.2014.1744 24885917 PMC10938214

[9] Hurst M, Nesbitt A, Kadykalo S, Dougherty B, Arango-Sabogal JC, Ravel A. Attributing salmonellosis cases to foodborne, animal contact and waterborne routes using the microbial subtyping approach and exposure weights. Food Control. 2023;148:109636. 10.1016/j.foodcont.2023.109636

[10] Zhang S, Li S, Gu W, den Bakker H, Boxrud D, Taylor A, et al. Zoonotic source attribution of *Salmonella enterica* serotype Typhimurium using genomic surveillance data, United States. Emerg Infect Dis. 2019;25:82–91. 10.3201/eid2501.180835 30561314 PMC6302586

[11] Sodagari HR, Sahibzada S, Robertson I, Habib I, Wang P. Whole-genome comparative analysis reveals association between *Salmonella* genomic variation and egg production systems. Front Vet Sci. 2021;8:666767. 10.3389/fvets.2021.666767 34322531 PMC8311177

[12] Munck N, Leekitcharoenphon P, Litrup E, Kaas R, Meinen A, Guillier L, et al. Four European *Salmonella* Typhimurium datasets collected to develop WGS-based source attribution methods. Sci Data. 2020;7:75. 10.1038/s41597-020-0417-7 32127544 PMC7054362

[13] Munck N, Njage PMK, Leekitcharoenphon P, Litrup E, Hald T. Application of whole-genome sequences and machine learning in source attribution of *Salmonella* Typhimurium. Risk Anal. 2020;40:1693–705. 10.1111/risa.13510 32515055 PMC7540586

[14] Lupolova N, Dallman TJ, Holden NJ, Gally DL. Patchy promiscuity: machine learning applied to predict the host specificity of *Salmonella enterica* and *Escherichia coli.* Microb Genom. 2017;3:e000135. 10.1099/mgen.0.000135 29177093 PMC5695212

[15] Richardson LC, Bazaco MC, Parker CC, Dewey-Mattia D, Golden N, Jones K, et al. An updated scheme for categorizing foods implicated in foodborne disease outbreaks: a tri-agency collaboration. Foodborne Pathog Dis. 2017;14:701–10. 10.1089/fpd.2017.2324 28926300 PMC6317073

[16] Collins JP, Shah HJ, Weller DL, Ray LC, Smith K, McGuire S, et al.; Centers for Disease Control and Prevention. Preliminary incidence and trends of infections caused by pathogens transmitted commonly through food—Foodborne Diseases Active Surveillance Network, 10 US Sites, 2016–2021. MMWR Morb Mortal Wkly Rep. 2022;71:1260–4. 10.15585/mmwr.mm7140a2 36201372 PMC9541031

[17] Tolar B, Joseph LA, Schroeder MN, Stroika S, Ribot EM, Hise KB, et al. An overview of PulseNet USA databases. Foodborne Pathog Dis. 2019;16:457–62. 10.1089/fpd.2019.2637 31066584 PMC6653802

[18] Breiman L. Random forests. Mach Learn. 2001;45:5–32. 10.1023/A:1010933404324

[19] Coppersmith D, Hong SJ, Hosking JRM. Partitioning nominal attributes in decision trees. Data Min Knowl Discov. 1999;3:197–217. 10.1023/A:1009869804967

[20] Janitza S, Celik E, Boulesteix A-L. A computationally fast variable importance test for random forests for high-dimensional data. Adv Data Anal Classif. 2018;12:885–915. 10.1007/s11634-016-0276-4

[21] Wright MN, Ziegler A. ranger: a fast implementation of random forests for high dimensional data in C++ and R. J Stat Softw. 2017;77:77. 10.18637/jss.v077.i01

[22] Arnold M, Smith RP, Tang Y, Guzinski J, Petrovska L. Bayesian source attribution of *Salmonella* Typhimurium isolates from human patients and farm animals in England and Wales. Front Microbiol. 2021;12:579888. 10.3389/fmicb.2021.579888 33584605 PMC7876086

[23] Beshearse E, Bruce BB, Nane GF, Cooke RM, Aspinall W, Hald T, et al. Attribution of illnesses transmitted by food and water to comprehensive transmission pathways using structured expert judgment, United States. Emerg Infect Dis. 2021;27:182–95. 10.3201/eid2701.200316 33350907 PMC7774530

