# Supplementary material for: Attribution of Salmonella enterica to Food Sources by Using Whole-Genome Sequencing Data
Source: Emerg Infect Dis. 2025 Apr;31(4):783–90. doi: 10.3201/eid3104.241172 (PMC11950287; doi:10.3201/eid3104.241172)
Supplement: Appendix 2 — Additional information for attribution of Salmonella enterica to food sources by using whole-genome sequencing data. [file 24-1172-Techapp-s2.pdf]

Article DOI: https://doi.org/10.3201/eid3104.241172

EID cannot ensure accessibility for supplementary materials supplied by authors. Readers who have difficulty accessing supplementary content should contact the authors for assistance.

Attribution of *Salmonella enterica* to Food Sources by Using Whole-Genome Sequencing Data

Appendix 2

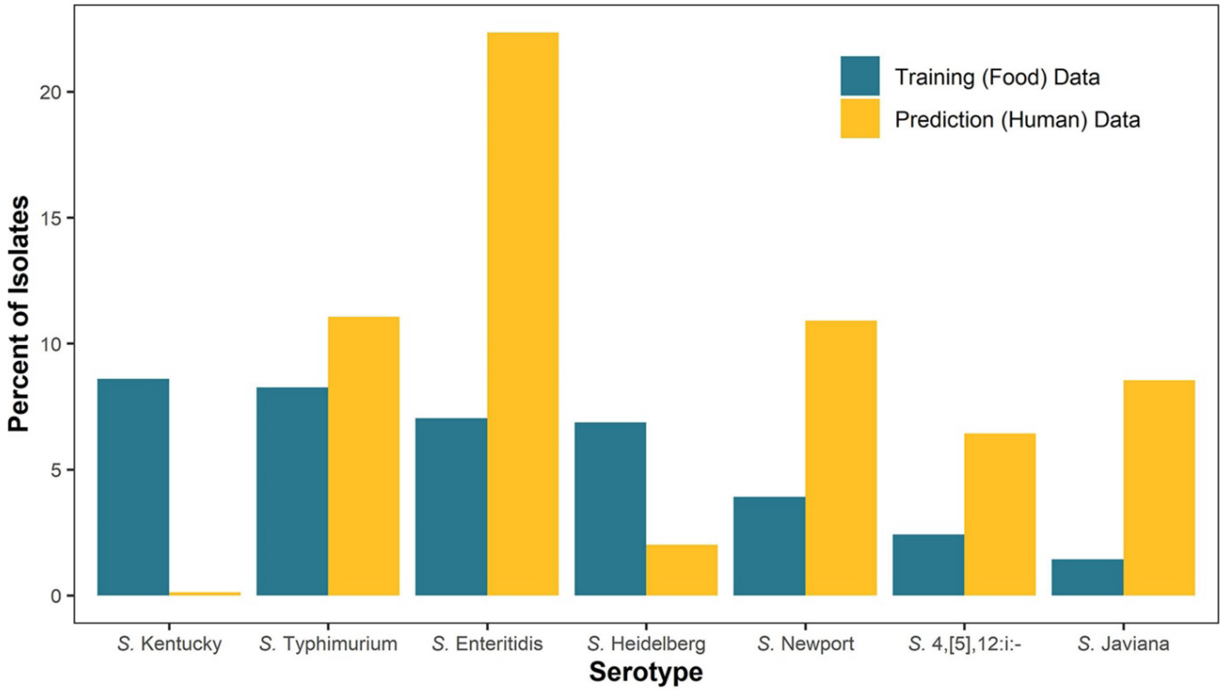

Appendix 2 Figure 1. Proportion of *Salmonella* isolates by serotype for the isolates collected from single food sources and used to train the random forest model (N = 18,661; blue) and isolates collected from humans with salmonellosis and used for model prediction (N = 6,470; yellow).

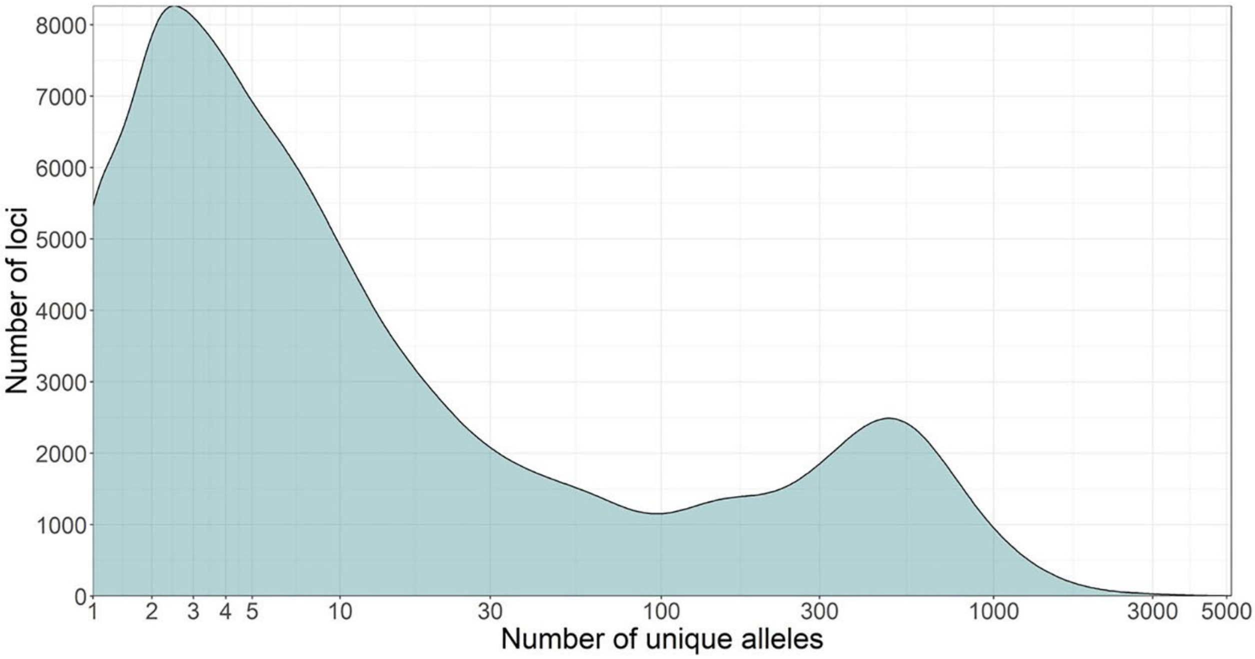

Appendix 2 Figure 2. The distribution of the number of unique alleles per locus among *Salmonella* isolates from a single food category used as training data in the random forest model (N = 18,661). The y-axis shows the number of loci that contain a given number of unique alleles, with missingness included as its own level.

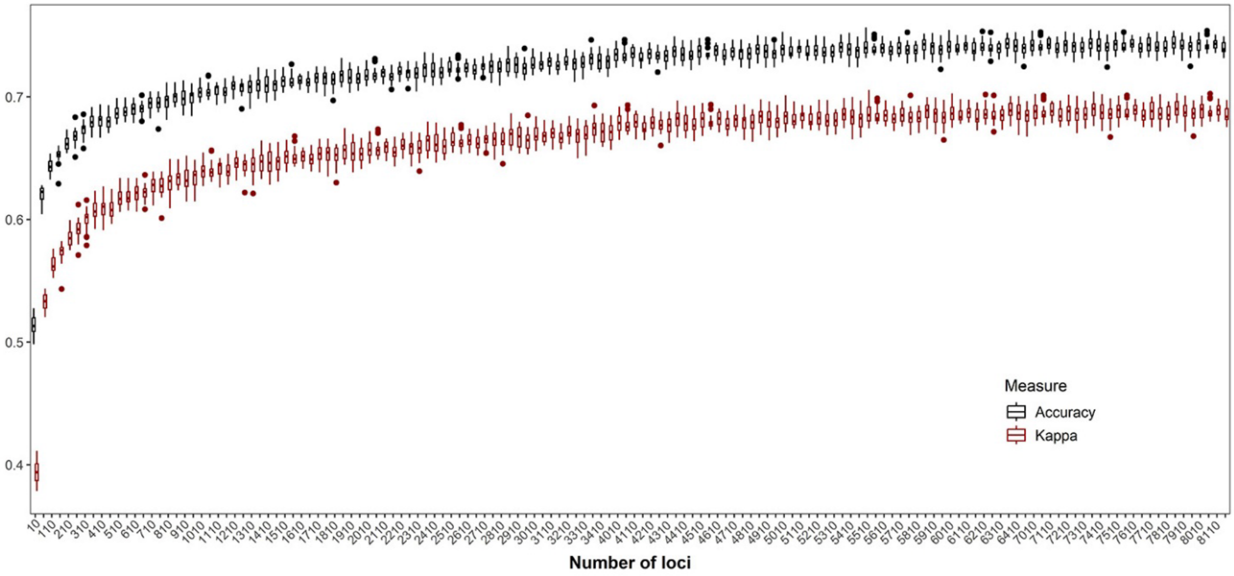

Appendix 2 Figure 3. Distribution of the out-of-bag accuracy (black) and kappa (red) when varying the inclusion of the number of top loci in the random forest ten times for each number of loci, in increments of 50 (N = 18,661). In this process, only loci with >1% non-missing data were included (8,143 loci). The median accuracy and kappa were both maximized using the top 7,360 loci.

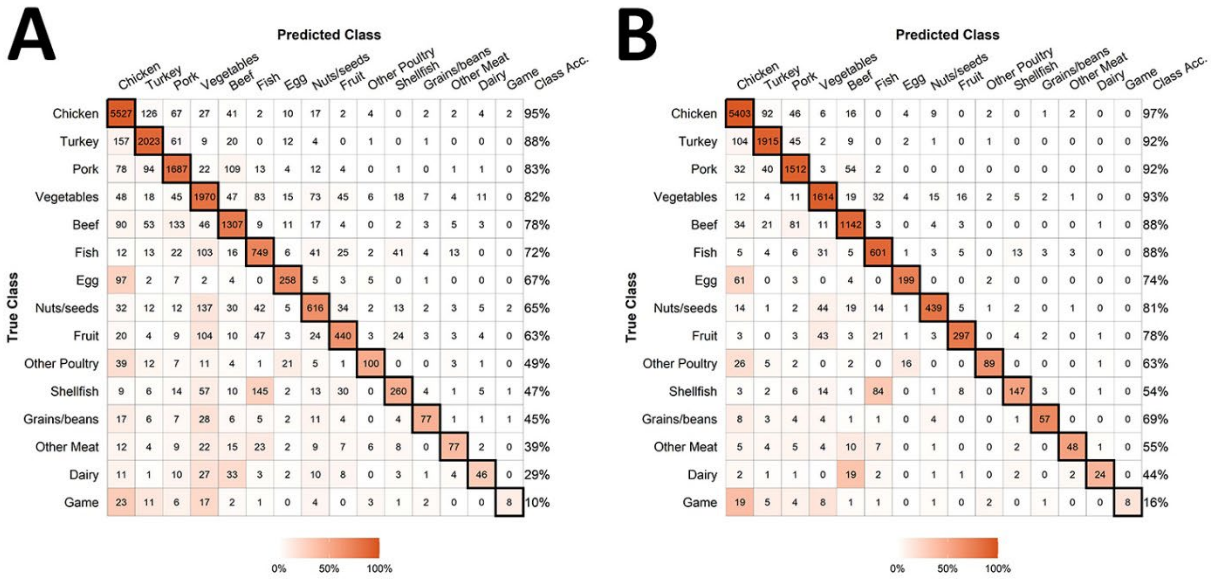

Appendix 2 Figure 4. Confusion matrix from the random forest model trained on *Salmonella* isolates collected from single food categories in the United States and other countries from 2003–2018 and 603 isolates collected before 2003. A) Confusion matrix for all *Salmonella* isolates from single food categories (N = 18,661). B) Confusion matrix from the random forest model for *Salmonella* isolates from single food categories with a maximum predictive probability of >0.50 (n = 14,888).

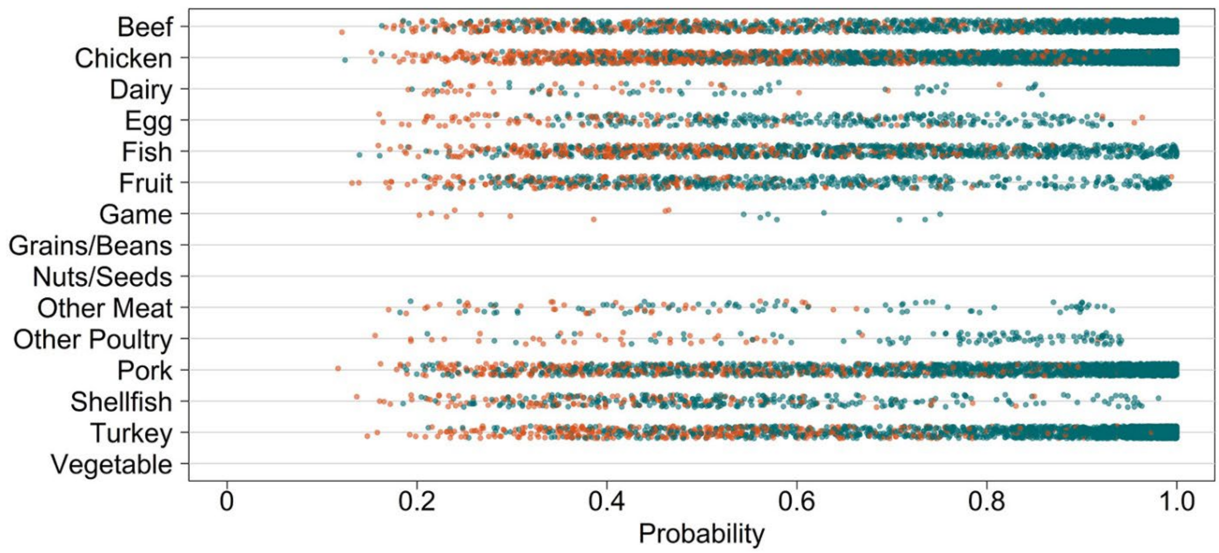

Appendix 2 Figure 5. The predicted probability (x-axis) for a given food category (y-axis) for each isolate used to train the random forest model (N = 18,661). Orange indicates the isolate was misclassified by the model for the given food category, while teal indicates the isolate was correctly classified.
